# Road and Transportation Lead to Better Health and Sustainable Destination Development in Host Community: A Case of China Pakistan Economic Corridor (CPEC)

**DOI:** 10.3390/ijerph182312832

**Published:** 2021-12-06

**Authors:** Liaqat Ali, Salim Khan, Syed Jamal Shah, Aman Ullah, Hina Ashraf, Mushtaq Ahmad, Abida Begum, Heesup Han, Antonio Ariza-Montes, Luis Araya-Castillo, Afed Ullah Khan, Muhammad Anas, Abdul Majid Khan

**Affiliations:** 1 Department of Sociology, University of Swabi Pakistan, Swabi 53561, Pakistan; aman@uoswabi.edu.pk (A.U.); hina@uoswabi.edu.pk (H.A.); mushtaqahmad@uoswabi.edu.pk (M.A.); abdulmajid@uoswabi.edu.pk (A.M.K.); 2 Riphah School of Leadership, Faculty of Management Sciences, Riphah International University Malakand Campus, Chakdara 18300, Pakistan; salimuom@yahoo.com or; 3 Department of Business Administration, Iqra University, Karachi 75850, Pakistan; jamal.shah@iqra.edu.pk; 4 School of Marxism, Northeast Forestry University, Harbin 150040, China; abidakhg@gmail.com; 5 College of Hospitality and Tourism Management, Sejong University, Seoul 05006, Korea; 6 Social Matters Research Group, Universidad Loyola Andalucía, Escritor Castilla Aguayo 4, 14004 Córdoba, Spain; ariza@uloyola.es; 7 Facultad de Economía y Negocios, Universidad Andrés Bello, Santiago 7591538, Chile; luis.araya@unab.cl; 8 Department of Civil Engineering, University of Engineering and Technology Peshawar (Bannu Campus), Bannu 28100, Pakistan; afedullah@uetpeshawar.edu.pk; 9 School of Applied Economics, Harbin University of Science and Technology, Harbin 150080, China; anashust1237@yahoo.com

**Keywords:** accessibility, CPEC, employment, health, road, transport, sustainable destination development

## Abstract

Road and transportation plays a vital role in the sustainable development and prosperity of the area. This study investigates the impact of road and transportation on the health of the host community and its sustainable destination development. Data were collected from the host community and were analyzed through factor analysis and structure equation modeling to evaluate the in-hand data of the structural relationship. It is revealed that road and transportation has a significant role in the improvement of health. Moreover, income mediates the effects of accessibility and employment on health. This study will help the authorities and policy maker to formulate policy regarding road and transportation that will improve health of the host community and its sustainable development. The study is limited to the seven districts of Hazara division and explores the societal aspect of CPEC on the host community, future researcher may investigate other regions and may select some other variables such as effect on GDP, per capita income, etc.

## 1. Introduction

Mobility play a pivotal role in the socio-economic diversity and sustainable development and social inclusion and lead to improvement in living standard, education, public health and work as a fuel for the development and prosperity of the region [1,2,3,4]. It is considered as an engine for economic growth [5]. It improves and sustains the regional economy, minimizes unemployment and poverty and improves the living standard of the host communities and makes it sustain [2]. The current research study focused upon the consequences of China–Pakistan Economic Corridor (CPEC) on the sustainable health sector of Pakistan. CPEC is a central part of the Mega Chinese project “Belt and Road Initiative (BRI)” started by the Chinese Government in 2013 [2]. The main purpose of the project is to link Asia with Europe and Africa under the vision of shared destiny [6]. The project’s major goal is to connect Asia with Europe and Africa through a common destiny vision [3]. BRI is a gigantic project that will link over 60 countries [6]. Early studies investigating CPEC have shown that it will not be beneficial only for both the nation but will also signifying other nations such as India, Iran, Afghanistan, Europe, and Africa [7]. CPEC is said to be the fate changer for the entire region, particularly for Pakistan [3].

Numerous studies examined the relationship mobility and sustainable economic growth using panel data [8,9] and by adopting research framework, which link roads networks with economic consequences and employment growth in the area using a partial least squares model [10]. It has been considered globally that roads and transportation are the source of income and employment opportunities as Dobbs [11] examined transport accessibility impacts on employment and revealed that women employment is more convenient due to accessible mobility. Moreover, studies in the United State of America (USA) using different evaluation technique and panel data explored that mobility has a dynamic role in the generation of employment opportunities [4,10,12]. Jiang, et al. [13], revealed significant impact of road and transportation on economic growth and productivity in China using structural equation model (SEM) approach and comprehensively consider the bi-directional relationship between multimodal transportation investment and economic development. Moreover, it has been revealed using a case study approach that transportation infrastructure plays an important role in the socioeconomic development of the area [14]. It is clear from the existing literature that roading and transportation has a great role in terms of employment and economic growth although the perceived impact of road and transportation on sustainable development of health has never been examined which motivate the author for the current study.

Road and transportation improve health and living standards of the host communities. Access to health services has a key role in the health system of any country and is considered one the main factors that encourages health [15]. The effect of roads and transport on income and employment have been investigated earlier. However, little attention has been given to the sustainable effects of roads and transportation accessibility on health, which motivated the authors for the current study. The main aim of this study is to investigate the sustainable effects of CPEC on health of the Pakistani people through improvement of road accessibility and employment opportunities, which has never been investigated earlier. Moreover, this study explores the mediating effect of income on accessibility and employment. The current study is an attempt to addresses the following questions. Firstly, to quantify the sustainable impacts of CPEC on health of the local dwellers. Secondly, to explore the mediating role of income among accessibility, employment, and health. Theoretical Background, Literature Review and Conceptual Framework.

Roads and transportation generate employment opportunities in the area, while roads access is key factor that trigger income, increase employment opportunities and dampen poverty in the area [14]. Kenyon, et al. [16] revealed that access to basic amenities of life has a key role in social exclusion while lack of roads infrastructure is a main hurdle in sustainable employment opportunities. Absence of transport infrastructure or poor roads is one of the causes for social exclusion [17]. Eberts [18] investigated that public infrastructure has a significant sustainable role in the economic growth of the area, they added that an increase in investment in transportation leads to huge economic output. Public infrastructure works as a tool for regional development as Porter [1] explored that roads infrastructure play a key role in the development and improvement of income and living standard in sub-Saharan Africa. Similarly, Rammelt and Leung [19] examined roads and transport infrastructure and their consequences on employment and productivity (income), they revealed that roads access increases local income as agriculture product can easily reach to market and sell their yield at good prices. Transport infrastructure also affects the health of the local residents, as lack of access to hospitals and healthcare center trigger mortality and morbidity ratio [20]. It is well-founded in the existing literature that lack of transport infrastructure deprived resident’s access to basic amenities of life, employment and other services. According to theory of accessibility access to basic amenities such as electricity improves education, income, and health of the region. This study revokes the work of Bridge and add perceived employment in the framework. Moreover, the widely used theory to investigate the host community perception is social exchange theory (SET) [21] and SET also make base and theoretical model for investigating and understanding the individual and community property [22]. Moreover, ref. [23] has been revealed that SET recommend that individuals can obtain responses via reciprocal base. Therefore, based on SET, the same is in the case of CPEC that if local community perceives that they are beneficial of the project they will participate. Furthermore, many attitudes-based investigation in [24] and [25] has already used SET. In this study, SET were used to look at the significance and response of the host community to CPEC development. SET asserts that, in the framework of the CPEC, the local community is likely to contribute to CPEC projects if they benefit from them. Finally, the conclusion in SET is that the host community will engage in and support the development of the CPEC if its citizens recognize the benefits and importance of the CPEC in the community’s and its development’s best interests, i.e., health access to local dwellers of the host community. In lens of accessibility theory and social exchange theory, this study investigates how transport infrastructure sustains and increases income and improves the health and living standard of the area. Roads play a vital role in the improvement and sustainable development of health. Health facilities are a primary need for an individual and can be defined as the aptitude of individuals to avail proper, reasonable and excellent medical services whenever they need it [15]. The health system of any country considered one of the main factors that encourage health [15]. it provides an easy access to hospital and health care center as Asomani-Boateng, Fricano and Adarkwa [14] reported a considerable increase of patients visit to medical and health care center due to transport infrastructural accessibility. It is believed that transport infrastructure provides convenient access to basic amenities. The consequences of roads and transportation on health are very significant in remote areas [15,26,27]. Similarly, Wagstaff [28] discovered that 10% increase in distance to medical facility trigger 2% mortality. A number of studies carried out on road and transport accessibility to health care center in different developed and developing countries [29]. In rural areas, unavailability of roads and transports are the main barriers toward health [30,31]. It is obvious from the literature that transport infrastructure is very useful for the local people’s health [31]. Kanuganti, Sarkar, Singh and Arkatkar [15] revealed that networks of roads play an important role in providing access to health care services. Similarly, transport accessibility centralizes health services and other basic facilities for the common people, while the absence of reasonable and accessible transport encourages ignorance of host communities from health care centers, training and academics opportunities [16]. Furthermore, Porter [1] revealed that roads and transports have a key role in the development and improvement. Based on the existing literature this study hypothesis that:

**Hypothesis** **1** **(H1).** *Perceived accessibility has a positively effects on health of the host community.*

**Hypothesis** **1a** **(H1a).** *Income mediates the positive effect of accessibility on health of the local residents.*

Roads and transportation generate many employment opportunities in the host communities, which directly affect the health of an individual and their families. Lankila, et al. [32] reported that job loss cause reduction in income, which affects individual and their family health. Similarly, existing literature suggests that lack of employment directly affects mortality and poor health [33]. Besides, Lee and Kim [34] discovered that employment play an important role in the health of an individual and his family. They further added that retirement negatively effects employs’ health. In this regard, Stuckler, Basu, Suhrcke, Coutts and McKee [33] revealed that lack of employment and low-income increase suicide rate. Employment significantly minimizes depression, stress and frustration [35]. Many studies explored that unemployment caused alcohol, cannabis use and causes many diseases which destroy health, while in Sweden unemployment makes alcohol consumption double, which badly affects health [36]. Similarly, Stuckler, Basu, Suhrcke, Coutts and McKee [33] discovered that 1% increase in unemployment trigger 0.79% suicide rate, while USD 10 invested reduces the unemployment effect on the suicide rate at 0.38%. Good job and excellent working environment make individual life prosperous and healthy [34]. The existing literature summarizes that employment has a significant impact on income while income directly affects the health of an individual. This study hypothesis that:

**Hypothesis** **2** **(H2).** *Perceived employment in CPEC has a direct positive effect on health of the host community.*

**Hypothesis** **2a** **(H2a).** *Income from CPEC mediates the positive effect of employment on health on local dweller.*

Road and transports have a significant impact on the economy of the country [37]. The main role of every road and transportation project is economic growth [38]. It improves productivity and increases income by reducing shipping charges, providing easy access to the market, availability of raw materials and time-saving. Transport infrastructure generates a lot of different employment opportunities, which boost the economy of the host residents and help an eliminating poverty from the area [39]. DETR [40] explored that 52% job searcher noted that lack of access to transport infrastructure are the main obstacle to job while 23% reported that poor road is one of the main barriers to getting a job, which negatively affects income. Roads and transportation enable the area for construction of health care center and make it easy for further construction of basic services such as schools, banks, etc. [41]. Porter [1] revealed that roads infrastructure exerts a significant effect on income of sub-Saharan Africa and is considered an important tool for eradicating poverty from the area. It makes possible to link farms to market and people to people, which fuels the local income [42]. It is one of the main sources that increase local income as agriculture products can be easily and safely shifted to market with minimum cost and farmers can sell their yield at good prices in a market [19,41]. Transport infrastructure minimizes 90% of shipping costs, which directly increases the income of the local people [43]. Moreover, existing literature suggests that roads infrastructure considerably increase land value [44]. Furthermore, Yu, De Jong, Storm and Mi [9] revealed that roads and transportation significantly affect productivity, while Allen, et al. [45] revealed that transport infrastructure has a vital role in economic growth of the region. It is summarized that roads and transportation has a positive impact on income [12,13,46,47,48,49,50]. Based on the existing literature, this study hypothesized that:

**Hypothesis** **3** **(H3).** *Perceived accessibility has a direct positive effect on income of the host community.*

Employment is one of the main sources of income around the world, while transport infrastructure plays a key role in the employment generation and business activities, which directly affects income. The main purpose of every road and transport infrastructure is the economic growth and employment opportunities [38], transport infrastructure directly affects income of the host communities. While employment plays a tremendous role in the income of an individual and family in the ribbon areas [51]. While lack of employment and business opportunities lead to weak and poor futures with low incomes, which sometimes is insufficient to cover basic needs [52]. Similarly, employment loss reduces 10 to 15% annual income of a family [53,54]. While, Starkey, et al. [55] revealed that road and transport infrastructure trigger income of the local people up to 25%. Roads and transportation are the main cause source that creates hundreds of employment opportunities and different business activities which engaged the local people and improve their income and living standards. As Egger and Etzel [56] state, unemployment leads to a poor living standard. Based on the existing literature, it is concluded that employment has a positive effect on the income of an individual and family. This study hypothesis that:

**Hypothesis** **4** **(H4).** *Perceived employment has a direct positive effect on income of the host community.*

Income is one of the main sources that fulfil all the needs of an individual and ranked their living standard in the society. Similarly, income plays a key role in better health of a person and their families and has a causal relationship with health while low income is one of the barriers towards access to standard health [26,31]. High investment in health offers good health, while low income has a negative relationship with access to health and willingness of an individual to visit healthcare center [57]. Hessel, et al. [58] state that financial fluctuation led to worse health and suicide were witnessed in Europe due to economic loss. Similarly, Job loss through economic crises discourages access to health care center [59]. Stuckler, Basu, Suhrcke, Coutts and McKee [33] discover that lack of employment and income cause stress and frustration which led to increase in suicide. Similarly, Simou and Koutsogeorgou [60] revealed that income strongly affects health and health care of an individual. Moreover, Catalano, et al. [61] explored that 15% to 30% depression and anxiety symptom increased due to job economic loss. They further added that financial decline trigger mortality rate, increase stress and psychological sickness. Similarly, Lee, et al. [62] reported that income loss significantly increased depression in Hong Kong. Income can worsen or better the health and wellbeing of an individual [63]. It is clear from the existing literature that income directly affects the health of a person and his family. This study hypothesizes that:

**Hypothesis** **5** **(H5).** *Perceived income has a direct positive effect on the health of the host community.*

**Figure 1:**
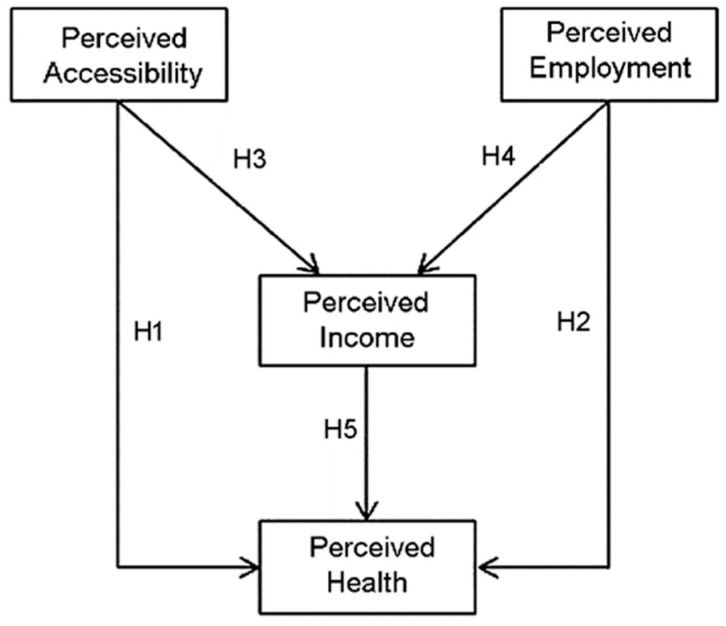
Hypothetical Model.

The hypothetical model of the study is shown in Figure 1.

## 2. Materials and Methods

To achieve the objective of the study, a comprehensive questionnaire was constructed with the help of a panel of professors, which are experts in the current research area including transportation, management and sociology. By following the work of Kim et al. [64], a list of observed variables were collected from the different studies which are related and appropriate for our study [2,3,21,65,66,67,68,69,70,71,72,73,74,75,76,77,78,79,80,81]. The wordings of these questions were changed according to the objective of the current study. Each professor was asked to examine the clarity, simplicity, wording and selection of the most appropriate question, which accomplishes the objective of the study. The panel suggests some modification for the clarity and understanding of respondents and based on the mutual consensus the most appropriate items were selected. After suggested modifications, the revised version was proceeded further. The questionnaire consisted of two main sections: demographic features and perceived impact of CPEC. Five-point Likert scales were used for measuring the response of the respondents.

All the indicators were observed by the panel and suggest the best fit for the accomplishment of our objective of the study. A pilot study was carried out with 63 questionnaires to find the complexity to respondents and their level of understanding. The validity and reliability of the questionnaire was evaluated through Cronbach’s alpha, mean and standard deviation of the in hand data. The panel reviews the finding of the instrument and gives some more suggestions based on the pilot study for more simplicity and clarity to respondents. After evaluation and modifications, perceived accessibility was assessed with nine-items, employment nine items, perceived income eight items [2], while perceived health was assessed with nine items (see Appendix A).

The present study was carried out in the seven districts of Mansehra, Bata Gram, Haripur, Upper Kohistan, Lower Kohistan, Torghar and Abbottabad of Hazara Division, Khyber Pakhtunkhwa (KPK), Pakistan. For the appropriateness of sample size of the respondents, the recommendation of [82] were followed that minimum ten respondents should be selected for each items. The questionnaires were distributed in 720 adult respondents in the study area and received 505 valid and useable responses (70.13% response rate). Data were analyzed in three main steps exploratory factor analysis (EFA), confirmatory factor analysis (CFA) and structure equation modeling (SEM). EFA was performed for dimension reduction, purification and identification of construct and were evaluated grounded on Kaiser–Meyer–Olkin Measure (KMO) of sampling adequacy and Bartlett’s test of sphericity (BTS). Factors were identified based on eigenvalue greater than one and pattern matrix loading above 0.4. The construct reliability analysis was performed using Cronbach’s alpha. CFA was used to factor structuring, validation and effectiveness of the questionnaire. For acceptability, a number of model fitness levels were assessed including normed Chi square (χ/df), comparative fit index (CFI), Tucker–Lewis fit index (TLI), root mean square error of approximation (RMSEA) and the standardized root mean squared residual (SRMR), while SEM was used to examine the structural relationship between observed and latent variables and their estimations.

## 3. Results

The demographic features of the respondents are demonstrated in Table 1. Out of the total respondents, 66.3% male and 33.7% were female respondents, 57.4% respondents were single while 42.6% were married. Moreover, this table indicated that 47.1% respondents were 16-year education, 39.8% respondents were 18 year of education while 8.1% respondents were Ph.D. Furthermore, the in hand data indicated that joint family system is the dominant family system in the study area as 73.3% respondents were living in the joint family system.

Evolving EFA the BTS was significant at *p* < 0.000, KMO measure of sampling adequacy shows good sample size of 0.924 the pattern matrix were above 0.4, explained 58.78% variance and four factors were identified as expected. Construct reliability analysis was performed using Cronbach’s alpha which explored that perceived accessibility (nine items, α = 0.90), perceived employment (nine items, α = 0.94), perceived income (eight items, α = 0.92) and perceived health (nine items, α = 0.91). The Cronbach’s alpha for all the variables was above the threshold value of 0.7 [83] as presented in Table 2. No cross loading was witnessed which proves the discriminant validity of the items. Convergent validity was assessed and the factors were verified. The findings of EFA indicated that the sample size is good; the factors were uncorrelated and the in hand data were suitable for further analysis [84].

### 3.1. Confirmatory Factor Analysis

CFA was carried out with four latent variables and 35 observed variables to check its validity and unidimensionality. The model was significant and good fit as normed Chi-square value (χ^2^/df) = 2.02 is under the threshold value <0.3 [85], SRMR = 0.037 (<0.08; [82], RMSEA = 0.045 (<0.08; [86], CFI = 0.95 and TLI = 0.95 as exhibited in Table 3. Moreover, it was observed that all the standardized factor loading was above the threshold value of 0.7 [87]. Average variance extracted (AVE) and construct reliability (CR) was observed to check the convergent validity and reliability of the measurement model. The AVE values ranged from 0.51 to 0.63 which are above the critical value >0.5 [88] and based on the inter-factor correlation. The entire factor has discriminant validity as all the values <0.85 [82] as shown in Table 4. The result summarizes that according to [82,86,88,89] recommended values for CR, AVE, convergent and discriminant validity our model is fit for the generalization and assessing factors.

### 3.2. Structural Equation Modeling

**Figure 2:**
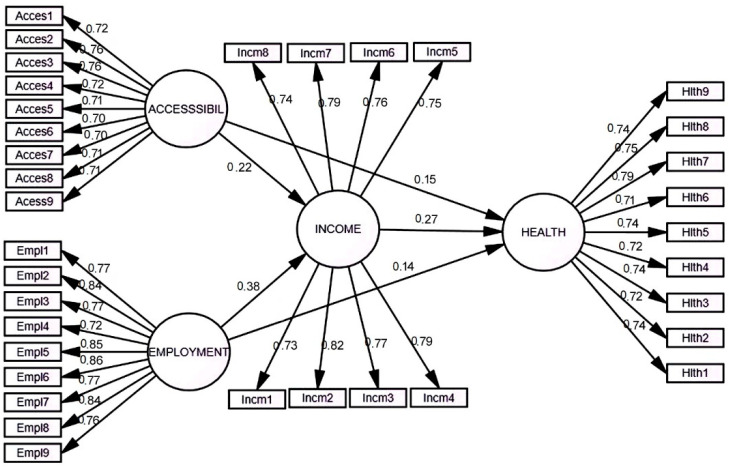
SEM Result.

SEM was carried out with five direct paths between endogenous and exogenous variables to find the relationship of observed and latent variables. Perceived accessibility and employment were selected as exogenous variables, while perceived income and health were selected as endogenous variables as indicated on Figure 2. Our findings suggest that the overall model was good and acceptable as we have a good model fit, *t*-values and standardized beta, which cross the critical values: Chi-square χ^2^/df = 1.976, RMSEA = 0.044, SRMR = 0.037, CFI = 0.956 and TLI = 0.952. The structural relations were observed and found that it had a *H1* standardized factor loading 0.261, *t*-value = 5.16, *p*-value < 0.001. This means that perceived accessibility has a direct significant positive sustainable effect on the health of the host community. The *H2* standardized factor loading was 0.286, *t*-value = 5.76, *p*-value < 0.001. This means that perceived employment has a significant direct positive sustainable effect on the health of the host community. The *H3* standardized factor loading was 0.216, *t*-value = 4.55, *p*-value < 0.001. This indicates that accessibility has a direct positive sustainable effect on the income of the local residents. The *H4* standardized factor loading was 0.382, *t*-value = 7.70, *p*-value < 0.001. These results stated that perceived employment has a significant direct positive effect on income of the local residents. The *H5* standardized factor loading was 0.274, *t*-value = 4.98, *p*-value < 0.001. Based on results, income has a sustainable direct positive significant influence on the health of the local dwellers.

### 3.3. Mediating Effect

The mediatingeffect was tested and examined that income mediates the positive effect of perceived accessibility on perceived health as supported by our result β = 0.149, *p* < 0.05, and *t*-value = 3.03. Thus, the hypothesis, *H1a*, has been accepted. Moreover, according to Baron and Kenny [92] an approach of income partially effects perceived accessibility on perceived health. Furthermore, the mediating effect of perceived income between perceived employment and perceived health (*H2a*) was observed and found that perceived income mediates the positive relationship between perceived employment and perceived health as supported by our findings that *p* < 0.05, β = 0.135, *t*-value = 2.63. According to Baron and Kenny [92], approach income partially mediates the effect of employment on health. As shown in Table 5.

## 4. Discussion

Roads accessibility is a key factor to improve employment opportunities, boost income and dampen poverty and crime [39,93]. The current study found that roads and transportation have a sustainable significant impact on the health of the host community and the finding is consistent with the recent study of Asomani-Boateng, Fricano and Adarkwa [14]. According to them, transport infrastructure accessibility plays a tremendous role while offering access to health care services and effective in coping health issues [31]. This study revoked the bridge theory and the results are consistent, according to this theory accessibility improve health, education and income of the local of the area [2,3]. This study explored that after providing road access a considerable increase of patient witnessed in the hospital and health care center. Transport infrastructure is the essential part of health and play a significant role in the sustainable development of health specifically in remote areas [27]. Roads centralized medical services for public and give easy access to these center and fulfill the basic need of life [16]. Similarly, it helps the common people to offer excellent health services [1]. CPEC will provide an easy, reasonable and speed access to hospitals and different health care centers which will directly affect the health of the host communities. Moreover, establishment of new hospital and health care center is also the part of CPEC which led to standard health and sustainable development of the local dwellers.

This study found that perceived employment has a direct positive effect on the health of the host community. Our results is inlined with the finding of Lankila, Näyhä, Rautio, Rusanen, Taanila and Koiranen [32]. They revealed that roads have a causal relationship with the health of the host communities. It is expected that CPEC will boost employment opportunities in the area, which directly improves the health of the host community [34]. Employment keeps an individual and his family healthy and happy as it is the main source of income all over the world, which fulfills the basic needs of life [94]. Employment plays a key role in the sustainable development and improvement in health as unemployment leads to frustration and depression which ultimately led to suicide [33]. Similarly, lack of employment in youth led to high consumption of alcohol, frustration and depression [36]. CPEC will cover the unemployment and eliminate poverty in the region, which will improve the living standard of local peoples [94]. The unemployment ratio in Pakistan is high which is the mother of all social problem, while CPEC will help in minimizing unemployment as lack of job lead to suicide while the investment discourages this rate [33]. Roads infrastructure and employment play a significant effect on health, while excellent employment and high income keep an individual healthy [34]. It is expected that CPEC may generate millions of jobs in the country which indirectly or directly affect the health of the local people of Pakistan. Moreover, construction of some new hospitals and health care centers and improvement of already established hospitals is also a part of the CPEC, which may facilitate and led to sustainable development of the local people of the area.

Road and transportation have a strong positive significant impact on the economic growth. This study explored that perceived accessibility has a direct positive impact on the income of the host community in the study area. The findings of Agbelie [37] support our results that road and transport accessibility affect the income of the dwellers in the host communities. Road and transport accessibility provide an easy access to market and urban areas where a person can find good jobs and better income-generating opportunities, which boosts the income of an individual [14]. It offers an easy access to cities and markets, which minimize travel time and shipping cost, availability of input material for farmer boost the income of the local dwellers [41]. Roads accessibility is the main strategy for eradicating poverty and sustainable development of the area [1]. Similarly, CPEC will enhance the broken economy of Pakistan as and transportation is the backbone of the economy of an area [38]. CPEC will offer access to cities in many areas, as a large portion is passing through remote areas where agriculture products can easily reach to the market [14]. The farmers can sell their yield at good prices and purchase their input material at reasonable prices, which will improve the income of the local residents which directly improve the living standard of the local people [41], as transport infrastructure reduced shipping cost up to 90% which directly improve the income of the host communities [43]. Moreover, CPEC will improve the land value in the ribbon areas [44]. Furthermore, CPEC will improve regional economic growth as recent studies prove that transport infrastructure significantly affects regional economic growth [13,49,50]. Most of Pakistani people are living in rural areas and CPEC has the ability to generate different income and trade opportunities, which may boost the income and sustainable development of the local residents in Pakistan.

It is explored in this research that employment has a direct significant impact on the income of the people of the study area. Our results were supported by work of Kalil and Ziol-Guest [52], which revealed that employment directly affects the income of an individual. Transport infrastructure keeps an important role in employment growth and economic development. As recent literature explored, transport infrastructure had a significant impact on employment generation, which directly leads to income growth of the local people [13,38,49,50]. While Starkey, Tumbahangfe and Sharma [55] stated that roads and highways increase income of the local dwellers by 25% as it offers different activities in the area which contribute to increases in income. It is expected that CPEC will generate millions of jobs, which will directly improve the income and living standard of the local people of Pakistan.

Furthermore, this study explored that income has a direct positive effect on the health of the local residents in the study area. The results is consistent with the recent study Pickett and Wilkinson [63] as they revealed that income has a causal relationship with health, the more the income the better will be the health, while low-incomes negatively affects an individual and his family access to health. In many European countries, the 2008 financial crises badly affect people’s health due to stress and frustration many of them commit suicide [58]. Similarly, Stuckler, Basu, Suhrcke, Coutts and McKee [33] also concluded the same result that lack of employment and income caused stress and frustration, which badly affects health and triggers the suicide ratio [60]. As economic collapse and employment loss increased depression by 15 to 30% in the people [61]. CPEC has the ability to boost the income and sustainable development of the Pakistani people and the broken economy of Pakistan by providing easy access to different opportunities and generating different economic activities in the region which directly affect the health of the local people and their living standards. CPEC will not only affect Pakistan’s economy but it will also boost the economy of the whole region including Afghanistan and central Asian countries.

## 5. Conclusions

The main aim of this study was to investigate the consequences of CPEC on the health of the local dwellers of Pakistan. Two exogenous variables (perceived employment and perceived accessibility) and two endogenous variables (perceived income and perceived health) were used to address the research question of this study. This research revealed that perceived accessibility has a direct positive and sustainable effect on the health of the local people of Pakistan. The absence of healthcare services in rural areas is one of the main problems. Roads provide access to the basic amenities of life such as health, which discourages the mortality rate in the area. Lack of healthcare centers in the area compels dwellers to visit urban areas or nearby towns to avail the services which take too much time to commute to by walking. This study discovered that roads infrastructure generate employment opportunities which directly affect the health of the local people. Employment plays a significant role in the health of an individual and their family as employment is the main source of income in every field, which covers the basic needs such as health. CPEC will generate employment opportunities, which directly affect the health of the local residents in the study area. This study found that roads accessibility has a sustainable positive effect on the income of the local people. CPEC will enable the local residents to access different trade and business opportunities, which directly enhance the income of the local people which will improve the living standard of the Pakistani people. Moreover, this research found that employment has a direct positive effect on the income of the people. It is obvious that employment directly affects the income of the people. It is expected that CPEC will generate millions of jobs during and after construction, which will benefit the local people of Pakistan. Furthermore, it is explored in the study that income has a direct positive effect on the health of the local people of the study area. Moreover, this study discovered that income mediates the positive effect of accessibility and employment on the health of the local people. CPEC is a mega project that not only provides access to China for their trade, but it also provides access to the Pakistani people to different amenities of life such as health. It will enhance the broken economy of Pakistan and will develop the socio-economic status of the Pakistani people, which may improve living standards and sustainable development of Pakistanis.

This study dwells upon the importance of CPEC and their perceived impact on the health of the local people. Many people of Pakistan are resisting against the construction of CPEC regarding personal and political interests. This research will make aware to the local people how CPEC can improve their health, living standard and sustainable development in the near future. Policy makers and government authorities can take guidelines from this research for the sustainable development of policy regarding health improvement and related issues. This study is limited to the seven districts of KPK, while future scholars can find the effect on all the provinces of Pakistan, which make a detailed comparison of the results. Furthermore, this study is limited to the positive and sustainable developmental impact of CPEC on health—future scholars can focus on the negative impact of CPEC on the health of the local people.

## Figures and Tables

**Table 1:** Demographic Characteristic of the Respondents.

Characteristics	*N* = 505	Percentage	S.D
**Gender**			**0.473**
Male	335	66.3	
Female	170	33.7	
**Marital status**			**0.495**
Married	215	42.6	
Unmarried	290	57.4	
**Family structure of the respondents**			**0.503**
Joint	370	73.3	
Extended	37	7.3	
Nuclear	98	19.4	
**Education of the respondents**			**0.754**
10-year education	7	1.4	
12-year education	18	3.6	
16-year education	238	47.1	
18-year education	201	39.8	
Ph. D and above 18-years of education	41	8.1	

**Table 2:** Pattern Matrix, Communalities, Average Variance Extracted, Explained Variance and Standardized Loading.

Measurement *	Pattern Matrix	Standardized Loading	Cronbach’s Alpha	Explained Variance
**Accessibility**			**0.90**	**10.49%**
Acces1	0.784	0.717		
Acces2	0.807	0.760		
Acces3	0.755	0.756		
Acces4	0.716	0.720		
Acces5	0.702	0.708		
Acces6	0.684	0.704		
Acces7	0.687	0.703		
Acces8	0.699	0.705		
Acces9	0.707	0.708		
**Health**			**0.91**	**12.24%**
Hlth1	0.751	0.738		
Hlth2	0.713	0.720		
Hlth3	0.744	0.743		
Hlth4	0.694	0.722		
Hlth5	0.719	0.743		
Hlth6	0.687	0.711		
Hlth7	0.782	0.787		
Hlth8	0.809	0.749		
Hlth9	0.799	0.737		
**Employment**			**0.94**	**27.82%**
Empl1	0.732	0.766		
Empl2	0.813	0.840		
Empl3	0.738	0.772		
Empl4	0.674	0.724		
Empl5	0.898	0.850		
Empl6	0.876	0.856		
Empl7	0.736	0.766		
Empl8	0.902	0.841		
Empl9	0.839	0.761		
**Income**			**0.92**	**8.23%**
Incm1	0.697	0.735		
Incm2	0.840	0.818		
Incm3	0.824	0.770		
Incm4	0.806	0.786		
Incm5	0.709	0.753		
Incm6	0.746	0.756		
Incm7	0.795	0.790		
Incm8	0.742	0.744		

* = The column measurement is indicated with coded names of items for details refer to the Appendix A.

**Table 3:** Goodness of Fit for Measurement Model and Structure Model.

Goodness of Fit	Recommended Value (Reference)	Measurement Model and SEM Model Fit
χ^2^/df	<3.00 [85]	2.02
RMSEA	<0.100 [90]	0.045
SRMR	<0.080 [82]	0.037
CFI	>0.950 [86]	0.954
TLI	>0.950 [91]	0.949

RMSEA = root mean square error of approximation, SRMR = standardized root mean square residual, CFI = comparative FIT index, TLI = Tucker–Lewis index.

**Table 4:** Composite Reliability, Average Variance Extracted, and Inter-factor Correlation (*n* = 505).

	CR	AVE	Accessibility	Employment	Health	Income
Accessibility	0.907	0.519	0.720			
Employment	0.940	0.638	0.220	0.799		
Health	0.915	0.546	0.261	0.286	0.739	
Income	0.922	0.596	0.298	0.428	0.382	0.772

CR = construct reliability, AVE = average variance extracted.

**Table 5:** Hypothesis Testing.

Hypothesis	Relationship	Standardized Estimates	*t*-Value	Result
*H1*	Accessibility → Health	0.261 ***	5.16	Support
*H1a*	Accessibility → Income → Health	0.149 **	3.03	Support
*H2*	Employment → Health	0.286 ***	5.76	Support
*H2a*	Employment → Income → Health	0.135 **	2.63	Support
*H3*	Accessibility → Income	0.216 ***	4.55	Support
*H4*	Employment → Income	0.382 ***	7.70	Support
*H5*	Income → Health	0.274 ***	4.98	Support

*** = *p* < 0.001, ** = *p* < 0.05.

## Data Availability

Data is available and will be provided on request.

## Appendix A

**Perceived Accessibility**
1. CPEC will increase easy access to quality health care and hospitals (Access1)
2. CPEC will provide access of farmer to the big cities through building chain roads/routes (Access2)
3. CPEC provide access to quality education (Access3)
4. CPEC will provide access to the remote area (Access4)
5. CPEC will provide access to quality of public services—fire, police, etc. (Access5)
6. CPEC will reduce shipping cost and provide access to high-quality mode of transportation (Access6)
7. CPEC will reduce travel time to destination (Access7)
8. CPEC will provide good public transportation system (Access8)
9. CPEC will generate opportunity to meet people from other cultures (Access9)
**Perceived Employment**
1. CPEC will generate employment opportunities in the area (Empl1)
2. CPEC will create chances for a person to find a good job (Empl2)
3. CPEC will generate new business opportunities in the area (Empl3)
4. A subsequent decline in poverty is imminent in light of CPEC (Empl4)
5. Employment Wages will be better in CPEC jobs (Empl5)
6. CPEC will provide employment and skills for improved livelihood opportunities (Empl6)
7. More employment opportunities mean less crime (Empl7)
8. CPEC will minimize social problem due to employment (Empl8)
9. Career opportunities will be better than old job (Empl9)
**Perceived Income**
1. CPEC will increase the household income (Incm1)
2. CPEC will generate revenue in the local economy (Incm2)
3. Feeder roads along CPEC will increase the economic activities which lead to increase in income (Incm3)
4. CPEC will improve the economic conditions of the area (Incm4)
5. CPEC will improve living standard of the common people (Incm5)
6. CPEC will Cover Current energy crises in the country which lead to increase in family income (Incm6)
7. Long term, investments have positive economic consequences (Incm7)
8. CPEC would mitigate the poverty across its lines (Incm8)
**Perceived Health**
1. Essential of basic ammonites such as health are ensured in CPEC (Hlth1)
2. New health facilities are expected to be established under CPEC (Hlth2)
3. Under the CPEC health will be improved by introducing new, advanced tools and equipment (Hlth3)
4. Availability of electricity is essential for the functioning and delivery of health services (Hlth4)
5. Under CPEC better transportation networks may also contribute to easier access to health care (Hlth5)
6. CPEC will help to reduce different disease by access to clean, safe water and sanitation infrastructure (Hlth6)
7. Under the CPEC project health of the common people will be improved (Hlth7)
8. Availability of recreational facilities will improve health (Hlth8)
9. CPEC will increase noise level which causes to different health problem (Hlth9)
